# Validation Study of SNPs in CAPN1-CAST Genes on the Tenderness of Muscles (*Longissimus thoracis* and *Semimembranosus*) in Hanwoo (Korean Cattle)

**DOI:** 10.3390/ani9090691

**Published:** 2019-09-17

**Authors:** Hyo Jun Lee, Shil Jin, Hyoun-Joo Kim, Mohammad Shamsul Alam Bhuiyan, Doo Ho Lee, Soo Hyun Lee, Sung Bong Jang, Man Hye Han, Seung Hwan Lee

**Affiliations:** 1 Division of Animal and Dairy Science, Chungnam National University, Daejeon 305-764, Korea; gywns6298@naver.com (H.J.L.); wlstlf21@cnu.ac.kr (S.J.); joos0621@korea.kr (H.-J.K.); stonecold@daum.net (D.H.L.); lhyungm@cnu.ac.kr (S.H.L.); jsbng8615@gmail.com (S.B.J.); 2 Hanwoo Research Institute, National Institute of Animal Science, PyeongChang 232-956, Korea; hanmhh@korea.kr; 3 Department of Animal Breeding and Genetics, Bangladesh Agricultural University, Mymensingh 2202, Bangladesh; msabhuiyan@gmail.com

**Keywords:** single nucleotide polymorphism, μ-calpain, calpastatin, muscle, Hanwoo

## Abstract

**Simple Summary:**

For meat tenderness, single nucleotide polymorphisms (SNPs) in the μ-calpain (*CAPN1*) and calpastatin (*CAST*) genes have been reported to be associated with Warner-Bratzler shear force (WBSF) in different cattle populations, including Korean Hanwoo cattle. In this study, we validated the association of seven SNPs in *CAPN1* and *CAST* genes with meat tenderness in two different muscle cuts tenderness in the *Longissimus thoracis* (LT) and *Semimembranosus* (SM) muscles. Two SNPs in *CAPN1* and one SNPs in *CAST* genes showed association with WBSF of both muscle types. Furthermore, of twelve reconstructed haplotypes, six demonstrated significant associations with WBSF values. These findings may be one of the strong evidences that *CAPN1* and *CAST* gene mutations are strongly associated with WBSF. The information of significantly-associated SNPs and the resulted haplotypes could be utilized in the Hanwoo breeding program for further genetic improvement of tenderness traits.

**Abstract:**

Previous studies demonstrated that polymorphisms in the μ-calpain (*CAPN1*) and calpastatin (*CAST*) genes had significant effects on meat tenderness in different cattle populations. The aim of this study was to validate the potential association of seven single nucleotide polymorphisms (SNPs) harbored in these two candidate genes with meat tenderness in the *Longissimus thoracis* (LT) and *Semimembranosus* (SM) muscles. A total of 1000 animals were genotyped using TaqMan SNP genotyping arrays, and the meat tenderness of two muscle (LT and SM at 7 days post-slaughter) was assessed based on Warner-Bratzler WBSF (WBSF) testing. We observed significant associations of the *CAPN1*:c.580T>C, *CAPN1*:c.658T>C and *CAST*:c.1985G>C polymorphisms (*p* < 0.05) with the WBSF values in the LT and SM muscles. Additive effects of the C allele in *CAPN1*:c.580T>C and *CAST*:c.1985G>C were associated with an increase of 0.16 and 0.15 kg, and 0.08 and 0.26 kg WBSF in the LT and SM, respectively; *CAPN1*:c.658T>C had negative effects on the WBSFs. Furthermore, six reconstructed haplotypes demonstrated significant associations with WBSF values (*p* < 0.05). In conclusion, the significant associations identified between the SNPs in *CAPN1*, *CAST* and WBSF values could be utilized in marker-assisted selection programs in order to improve the beef tenderness of Hanwoo cattle.

## 1. Introduction

Tenderness is an important meat quality determinant in the beef industry, as consumers are willing to pay more for lean and tender meat [1]. Meat tenderness, the most highly researched palatability characteristic, is influenced by multiple factors, including marbling, juiciness, and flavor [2]. Compared to past trends, Korean consumers currently prefer highly marbled meat and exhibit a strong willingness to purchase tender meat that promotes the utilization of a system that ensures high-quality meat production [3]. To identify reliable gene markers that explain the large genetic and phenotypic variations in beef tenderness, an association analysis is required to validate previously tested gene markers and their subsequent effects in a large independent reference population [4]. For meat tenderness, SNPs in the *CAPN1* and *CAST* genes have been reported to be associated with WBSF in several cattle breeds [5,6,7,8].

*CAPN1/CAST* is an endogenous calcium-dependent proteinase system that mediates the proteolysis of key myofibrillar proteins during the postmortem storage of carcasses and cuts of meat at refrigerated temperatures. *CAPN1* is responsible for the breakdown of myofibrillar proteins and is associated with meat tenderness [9]. *CAST* inhibits μ- and m-calpain activity, which regulates postmortem proteolysis. The effects of DNA markers on these genes have been validated in many temperate breeds, tropical-adapted zebu, and tropical composites [4,10,11,12]. Genetic tests for meat tenderness that utilize SNPs in *CAST* and *CAPN1* have already been commercialized by Igenity TenderGENE (Merial Ltd., Atlanta, GA, USA) and GeneSTAR Elite Tender (Genetic Solutions Pty. Ltd., Brisbane, Australia). In addition, seven SNPs in *CAPN1-CAST* systems affecting meat tenderness were also identified in Hanwoo cattle [13]. However, the resulting effects of these gene markers need to be validated in a large reference population in order to commercialize a palatability prediction system. In this study, we validated the association of seven SNPs in *CAPN1* and *CAST* genes with meat tenderness in two different muscle cuts (LT and SM) in Hanwoo steers.

## 2. Materials and Methods

### 2.1. Animal and Phenotype Measurement

To validate *CAPN1-CAST* SNP effect, the DNA and Phenotypes datasets (n = 1000) was derived from samples collected from the Daegwallyeong Hanwoo Co. abattoir (Gangwon province, Korea). Animal Care and Use Committee approval was obtained for this study based on instructions for animal health and welfare outlined by the National Institute of Animal Science, Rural Development Administration (RDA), Korea. Management and feeding system were similar for the studied population. Calves weaned at 5 or 6 month and individuals were transferred by 10 per each pen. All calves were freely fed with a growing ration that consisted of hay and concentrate mixtures from 6 to 11 month of age. The percentage of TDN and CP were 70% and 15%, respectively. After that, steers were fed differently between early fattening period (12–15 mo), middle fattening period (16–21 mo) and final fattening period (22–29 mo). Individuals received concentrates and a rice straw-based ration with proportion in total feed of 2.5:1, 4.5:1 and 8:1 for each fattening period. Percentages of CP and TDN of the concentrates were 14% and 71%, 13% and 72% and 12% and 73%, respectively. All steers were slaughtered at 30 months of age and muscle samples (1.5 kg) were collected from the *Longissimus thoracis* (LT) and *Semimembranosus* (SM) muscles to measure Warner-Bratzler WBSF (WBSF) values. This half sib steer population (n = 1000) was derived from 120 progeny tested bulls and unrelated dams (2–10 progeny per bull). The WBSF values of two cooked steaks (LT and SM at 7 days post-slaughter) was measured according to the method of Wheeler et al. [14]. Steaks of 2.5 cm with an ~80 g weight were put into polyethylene bags. The bags were heated in a water bath at 80 °C for 30 min until the internal temperature of the steak reached 70 °C. After cooking, the meat samples were gradually cooled at room temperature for 30 min. Then, 1.27-cm diameter cores were removed from each meat steak parallel to the muscle fiber. WBSF values were measured with a Warner-Bratzler shear force attachment on an Instron Universal Testing machine (Instron Corporation, Canton, MA, USA). The operating parameters: load cell, 50 kg and crosshead speed, 200 mm/min. The summary statistics for the WBSFs of the two muscles are described in Table 1.

### 2.2. Genotyping

Three SNPs in bovine *CAST* and four SNPs in bovine *CAPN1* were genotyped using TaqMan SNP genotyping arrays designed by Applied Biosystems (LSK, Seoul, Korea) with a Bio-Rad real-time PCR [13]. Detailed information on the locations and positions of the markers is provided in Tables S1 and S2. The *CAPN1*:c.948G>C, which has a high LD relationship with *CAPN1*:c.580T>C, marker is included in commercial test panel of the Australian GeneSTAR tenderness [10]. The linkage disequilibrium (LD) and haplotype structure for four significant SNPs within the *CAPN1* gene were investigated in this study. The haplotypes for the 4 significant SNPs in the *CAPN1* gene were reconstructed using default parameters in PHASE and inspected by means of Heatmap plots obtained with Haploview to visualize recombination events and to define the length of haplotypes [15,16].

### 2.3. Statistical Analyses

A single-marker regression analysis was used to test for associations between the SNPs and WBSF values. Markers were assumed to be in linkage disequilibrium with quantitative trait loci in close proximity, and additive effects were evaluated. Statistical analyses were performed in ASReml [17]. To test for an association between each marker and the WBSF in different muscles, the following linear mixed regression model was utilized:

$$Y_{ijklm}=\mu +CG_{i}+b_{1}Sage_{j}+b_{2}IMF_{k}+\overset{7}{\underset{l=1}{\sum }}SNP_{l}+a_{m}+e_{ijklm}$$

where $Y_{ijklm}$ is the WBSF in different muscles, *μ* is a vector of the overall mean, $CG_{i}$ is a vector of the contemporary group for birth year, season, slaughter year, and birth place, $b_{1}$ is a regression coefficient, $Sage_{j}$ is a vector of the age of slaughter as a covariate effect, $b_{2}$ is a vector of Intramuscular fat (IMF) contents as a covariate, $SNP_{l}$ is a single-locus SNP genotype coded as 0, 1, and 2 as a covariate, $a_{m}$ is a vector of random polygenic effect $\sim N(0,A\sigma_{a}^{2}$); and $e_{ijklm}$ is a vector of random residual $\sim N(0,I\sigma_{e}^{2}$). The same statistical model was used for a haplotype association analysis. The percentage of the genetic variation explained by each significant SNP was calculated using the following formula [18].

$$\%Vg_{i}=100\times \frac{2p_{i}q_{i}a_{i}^{2}}{\sigma_{g}^{2}}$$

where $p_{i}$ and $q_{i}$ are the allele frequencies for the ith SNP estimated for the Hanwoo population, $a_{i}^{2}$ is the estimated additive effect of the ith SNP on the WBSF phenotypes at different muscle (LT and SM), and $\sigma_{g}^{2}$ is the REML estimate of the polygenic variance in WBSF at different muscle (LT and SM) in the commercial population.

## 3. Results and Discussion

The genotype and allele frequencies of selected seven SNPs from *CAPN1* and *CAST* genes are presented in Table S3. The estimated allele frequencies for each marker of this study differed largely with the previous findings of [13].

This dissimilarity might be due to the three times larger sample size included in the present study than previous study. We identified a strong association between the SNP markers and WBSFs in the two muscles; the results are summarized in Table 2. Interestingly, significant effects of *CAST*:c.1985G>C, *CAPN1*:c.580T>C and *CAPN1*:c.658T>C (*p* < 0.05) on the WBSF of both muscle types were observed. SNP variants of *CAPN1* have been reported to be associated with *B. taurus*-oriented crossbred populations [18,19] and a diverse genetic background consisting of *B. taurus, B. indicus*, and their reciprocal crossbreds [6,20]. These findings support our results, despite the variation in the SNP location observed across cattle populations. For example, the significantly associated *CAPN1*:c.948G>C SNP in the LT of *B. taurus* cattle [20] demonstrated insignificant associations in Hanwoo cattle that exhibited breed or population-specific SNP associations for this gene. In addition, the lack of association for all SNPs identified in our previous study of Hanwoo cattle [13] compared to our present results might be due to differences in the allele frequencies and sources of investigated samples (Table S3). In this study, we found *CAPN1* and *CAST* gene mutation to have significant effects on the WBSF measured in both muscles of Hanwoo, as shown in the previous study [6,7,8]. These findings may be one of the strong evidences that *CAPN1* and *CAST* gene mutations are strongly associated with WBSF across breeds.

**Figure 1:**
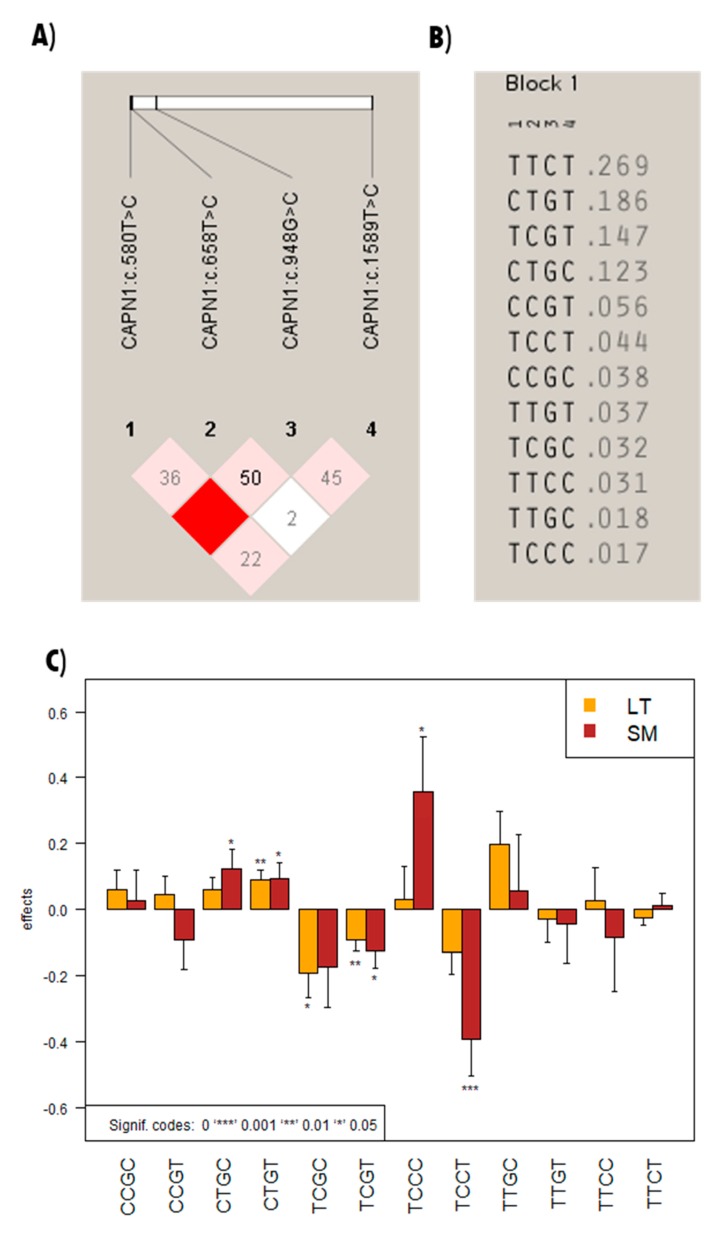
(**A**) Linkage disequilibrium pattern; (**B**) haplotype frequencies among the 4 SNPs in *CAPN1*; (**C**) haplotype effects on WBSF in different muscles in Hanwoo; The numbers at the top of panel (**B**) are equal to the numbers at panel (**A**) and those numbers represent each marker above.

Additive effects of the C allele of *CAST*:c.1985G>C and *CAPN1*:c.580T>C were associated with an increase of 0.15 and 0.16 kg WBSF, and of 0.26 and 0.08 kg WBSF in the LT and SM, respectively. Conversely, *CAPN1*:c.658T>C exhibited a negative effect on the WBSF in both muscle types (Table 2). The other *CAPN1*:c.948G>C SNP marker exhibited insignificant effects on WBSF, despite its strong linkage disequilibrium with the *CAPN1*:c.580T>C marker, which is the most significant marker associated with WBSFs in the LT and SM (Figure 1A). *CAPN1*:c.948G>C was referred to as *CAPN316*, which has been observed to be associated with WBSF in several studies [5,11,19].

In our study, twelve possible haplotypes were reconstructed for association analyses of *CAPN1* polymorphisms with WBSF; the results are presented in Figure 1B. Haplotype TTCT had the highest frequency (27%), followed by CTGT (19%), TCGT (15%), and CTGC (12%), while the other eight haplotypes (TCCC, TTGC, TTCC, TCGC, TTGT, CCGC, TCCT, and CCGT) had a frequency of 2% to 6%. Among them, six haplotypes had significant effects (*p* < 0.05) on WBSF. The second most frequent haplotype, CTGT (19%), increased the WBSF to 0.09 (*p* < 0.01) and 0.10 kg (*p* < 0.05) in the LT and SM, respectively (Figure 1C). However, haplotype TCGT (15% frequency) exhibited negative effects on WBSF to −0.09 (*p* < 0.01) and −0.12 (*p* < 0.05). These results correspond to the single marker analysis results that *CAPN1*:c.580T>C had a positive effect on WBSF and *CAPN1*:c.658T>C had a negative effect. Haplotype TCGC only showed significant effects on the WBSF in the LT, while three other haplotypes (CTGC, TCCC, and TCCT) had significant effects on WBSFs in the SM. The most significant effect (*p* < 0.001) was observed for haplotype TCCT, which decreased the WBSF in the SM to −0.39 kg. While two variants (*CAPN1*:c.580T>C, *CAPN1*:c.658T>C) exhibited significant effects on both muscle types, the affected muscle types seem to have changed depending on the haplotype structures. Interestingly, haplotype TCCC observed at a low frequency (2%) in the Hanwoo population had a beneficial effect (+0.35 kg) on the SM. The haplotype structure could change the effect of a single marker because the results were the opposite of the single marker analysis. The most frequent haplotype (TTCT) had insignificant effects on WBSF. In the future, the significant haplotype associations identified in our study can be utilized to resolve the issue reported in a previous study [13], in which no significant haplotype associations for *CAPN1* in Hanwoo cattle were observed. The haplotype analysis provided more explicit results compared to a single SNP found in a particular region of the genome. (Barendse, 2011) reported that the haplotypes accounted for 80% more phenotypic variance than individual SNP in *ADIPOQ*, *CAPN1*, *CXCR4*, *CEBPA* and *FASN*; this evidence of a robust association with intramuscular fat content, compared to single SNP analyses of *B. taurus* [21].

In some previous studies, no *CAPN1-CAST* markers were found to be associated with the WBSFs and causal effects in different populations [4,6,22,23]. During the validation phase, marker effectiveness may not be equal across populations, despite commercialization of the used markers. An unmatched validation study could result from SNP frequency variation, differences in marker-causative mutation linkage phases, genetic-by-nature interactions or epistasis, as well as sample size effects and the trait measurement method [22]. Above all, single-marker associations have been utilized in various livestock studies and have commercial impact on the improvement of meat quality traits. The significantly associated SNPs found in our study could be utilized for genetic improvement of the tenderness trait in Hanwoo populations, and thereby assist in the establishment of a beef palatability prediction system in Korea.

## 4. Conclusions

The present independent validation study confirmed the association of *CAPN1* and *CAST* with WBSF in LT and SM muscles from Korean Hanwoo cattle. The information of significantly associated SNPs and the resulted haplotypes could be utilized in Hanwoo breeding program for further genetic improvement of tenderness traits. For the confirmed associations, additional studies are encouraged to utilize these SNPs in marker-assisted selection programs.

## Figures and Tables

**Table 1:** Summary statistics of Warner-Bratzler shear force (WBSF), intra-muscular fat (IMF), and age at slaughter in *Longissimus thoracis* and *Semimembranosus muscle* of Hanwoo.

Traits	*Longissimus thoracis* (n = 1000)				*Semimembranosus* (n = 1000)			
	Mean	SD	Min	Max	Mean	SD	Min	Max
WBSF (kg)	3.97	0.86	2.15	6.92	6.65	1.14	3.4	9.88
IMF (%)	15.83	4.26	4.82	29.34	4.89	1.85	1.5	15.61
Age (months)	30.8	1.45	21.2	36.6				

**Table 2:** Association of *CAST/CAPN1* SNP alleles on WBSF measurements for two different muscle cuts.

Markers	Allele	MAF ^a^	WBSF (kg) in *Longissimus thoracis* at 7 Days				WBSF (kg) in *Semimembranosus* at 7 Days			
			Effects	F-Value	*p*-Value	%Var	Effects	F-Value	*p*-Value	%Var
*CAST*:c.182G>A (rs109727850)	A	0.41	−0.02 ± 0.04	0.33	0.561	0.03	−0.03 ± 0.05	0.22	0.631	0.04
*CAST*:c.1526A>G (rs109384915)	G	0.41	−0.06 ± 0.05	1.91	0.170	0.28	−0.04 ± 0.06	1.31	0.255	0.08
*CAST*:c.1985G>C (rs110914810)	C	0.08	0.15 ± 0.07	4.70	0.032 *	0.53	0.26 ± 0.09	8.57	0.004 **	0.99
*CAPN1*:c.580T>C (rs17872079)	C	0.40	0.16 ± 0.06	28.32	1.33 × 10^−7^ **	1.98	0.08 ± 0.07	10.71	0.001 **	0.31
*CAPN1*:c.658T>C (rs17872093)	C	0.34	−0.09 ± 0.06	9.57	0.002 **	0.59	−0.13 ± 0.07	10.31	0.001 **	0.76
*CAPN1*:c.948G>C (rs17872000)	C	0.36	−0.03 ± 0.06	0.43	0.508	0.07	−0.05 ± 0.07	0.31	0.574	0.12
*CAPN1*:c.1589T>C (rs17871051)	C	0.26	0.03 ± 0.04	0.65	0.420	0.06	0.07 ± 0.05	2.11	0.149	0.19

^a^ MAF indicates a minor allele frequency; *p*-value: 0 < ** < 0.01 < * < 0.05.

## Supplementary Materials

The following are available online at https://www.mdpi.com/2076-2615/9/9/691/s1. Table S1, Information on the genotyped SNPs, Table S2, Sequences of the Taqman probes used for SNP genotyping, Table S3, Genotype and allele frequencies for 7 selected SNPs in *CAPN1-CAST* Genes.

## References

[1] Shackelford S.D., Wheeler T.L., Meade M.K., Reagan J.O., Byrnes B.L., Koohmaraie M. (2001). Consumer impressions of Tender Select beef. J. Anim. Sci..

[2] Aberle E.D., Forrest J.C., Gerrard D.E., Mills E.W. (2001). Principles of Meat Science.

[3] Cho S.H., Kim J., Park B.Y., Seong P.N., Kim G.H., Jung S.G., Im S.K., Kim D.H. (2010). Assessment of meat quality properties and development of a palatability prediction model for Korean Hanwoo steer beef. Meat Sci..

[4] Van Eenennaam A.L., Li J., Thallman R.M., Quaas R.L., Dikeman M.E., Gill C.A., Franke D.E., Thomas M.G. (2007). Validation of commercial DNA tests for quantitative beef quality traits. J. Anim. Sci..

[5] White S.N., Casas E., Wheeler T.L., Shackelford S.D., Koohmaraie M., Riley D.G., Chase C.C., Johnson D.D., Keele J.W., Smith T.P.L. (2005). A new single nucleotide polymorphism in CAPN1 extends the current tenderness marker test to include cattle of Bos indicus, Bos taurus, and crossbred descent. J. Anim. Sci..

[6] Casas E., White S.N., Wheeler T.L., Shackelford S.D., Koohmaraie M., Riley D.G., Chase C.C., Johnson D.D., Smith T.P.L. (2006). Effects of calpastatin and μ-calpain markers in beef cattle on tenderness traits. J. Anim. Sci..

[7] Li Y.X., Jin H.G., Yan C.G., Seo K.S., Zhang L.C., Ren C.Y., Ji X. (2013). Association of CAST gene polymorphisms with carcass and meat quality traits in Yanbian cattle of China. Mol. Biol. Rep..

[8] Barendse W.J. (2009). DNA Markers for Meat Tenderness. U.S. Patent.

[9] Wheeler T.L., Koohmaraie M. (1994). Pre-rigor and post-rigor changes in tenderness of ovine longissimus muscle. J. Anim. Sci..

[10] Johnston D.J., Graser H.U. (2010). 2010. Estimated gene frequencies of GeneSTAR markers and their size of effects on meat tenderness, marbling, and feed efficiency in temperate and tropical beef cattle breeds across a range of production systems. J. Anim. Sci..

[11] Allais S., Journaux L., Leveziel H., Payet-Duprat N., Raynaud P., Hocquette J.F., Lepetit J., Rousset S., Denoyelle C., Bernard-Capel C. (2011). Effects of polymorphisms in the calpastatin and μ-calpain genes on meat tenderness in 3 French beef breeds. J. Anim. Sci..

[12] Curi R.A., Chardulo L.A.L., Mason M.C., Arrigoni M.D.B., Silveira A.C., de Oliveira H.N. (2009). Effect of single nucleotide polymorphisms of CAPN1 and CAST genes on meat traits in Nellore beef cattle (*Bos indicus*) and in their crosses with Bos taurus. Anim. Genet..

[13] Lee S.H., Kim S.C., Chai H.H., Cho S.H., Kim H.C., Lim D., Choi B.H., Dang C.G., Sharma A., Gondro C. (2014). Mutations in calpastatin and μ-calpain are associated with meat tenderness, flavor and juiciness in Hanwoo (Korean cattle): Molecular modeling of the effects of substitutions in the calpastatin/μ-calpain complex. Meat. Sci..

[14] Wheeler T.L., Shackelford S.D., Koohmaraie M. (2000). Relationship of beef longissimus tenderness classes to tenderness of gluteus medius, semimembranosus, and biceps femoris. J. Anim. Sci..

[15] Stephens M., Smith N.J., Donnelly P. (2001). A new statistical method for haplotype reconstruction from population data. Am. J. Hum. Genet..

[16] Barrett J.C., Fry B., Maller J., Daly M.J. (2005). Haploview: Analysis and visualization of LD and haplotype maps. Bioinformatics.

[17] Gilmour A.R., Gogel B.J., Cullis B.R., Thompson R. (2006). ASReml User Guide Release 2.0.

[18] Falconer D.S., Mackay T.F.C. (1996). Introduction to Quantitative Genetics.

[19] Page B.T., Casas E., Quaas R.L., Thallman R.M., Wheeler T.L., Shackelford S.D., Koohmaraie M., White S.N., Bennett G.L., Keele J.W. (2004). Association of markers in the bovine CAPN1 gene with meat tenderness in large crossbred populations that sample influential industry sires. J. Anim. Sci..

[20] Morris C.A., Cullen N.G., Hickey S.M., Dobbie P.M., Veenvliet B.A., Manley T.R., Pitchford W.S., Kruk Z.A., Bottema C.D.K., Wilson T. (2006). Genotypic effects of calpain 1 and calpastatin on the tenderness of cooked M. longissimus dorsi steaks from Jersey × Limousin, Angus and Hereford-cross cattle. Anim. Genet..

[21] Barendse W. (2011). Haplotype analysis improved evidence for candidate genes for intramuscular fat percentage from a genome wide association study of cattle. PLoS ONE.

[22] Gill J.L., Bishop S.C., McCorquodale C., Williams J.L., Wiener P. (2009). Association of selected SNP with carcass and taste panel assessed meat quality traits in a commercial population of Aberdeen Angus-sired beef cattle. Genet. Sel. Evol..

[23] McClure M.C., Ramey H.R., Rolf M.M., McKay S.D., Decker J.E., Chapple R.H., Kim J.W., Taxis T.M., Weaber R.L., Schnabel R.D. (2012). Genome-wide association analysis for quantitative trait loci influencing Warner–Bratzler shear force in five taurine cattle breeds. Anim. Genet..

