# Supplementary material for: Validation Study of SNPs in CAPN1-CAST Genes on the Tenderness of Muscles (Longissimus thoracis and Semimembranosus) in Hanwoo (Korean Cattle)
Source: Animals (Basel). 2019 Sep 17;9(9):691. doi: 10.3390/ani9090691 (PMC6770136; doi:10.3390/ani9090691)
Supplement: Supplementary file 1 [file animals-09-00691-s001.pdf]

animals

Supplementary Table S1. Information on the genotyped SNPs.

Gene	BTA [a]	SNP Name	Position	RefSNP Alleles	Recoding	Key reference
CAST	7	*CAST*:c.182A>G (rs109727850)	98485261	A/G (FWD)	GG = 0, AG = 1, AA = 2	
		CAST:c.1526T>C (rs109384915)	98554459	C/T (FWD)	AA = 0, AG = 1, GG = 2	
		CAST:c.1985G>C (rs110914810)	98566736	C/G (FWD)	GG = 0, CG = 1, CC = 2	
CAPN1	29	*CAPN1*:c.580A>G (rs17872079)	44067070	A/G (FWD)	TT = 0, CT = 1, CC = 2	
		CAPN1:c.658C>T (rs17872093)	44067234	C/T (FWD)	TT = 0, CT = 1, CC = 2	
		CAPN1:c.948G>C (rs17872000)	44069063	C/G (Gly316Ala)	GG = 0, CG = 1, CC = 2	Page et al. (2004)
		CAPN1:c.1589G>A (rs17871051)	44085642	A/G (Val530Ile)	TT = 0, CT = 1, CC = 2	Page et al. (2004)

[a] BTA indicates *Bos Taurus* autosom.

Supplementary Table S2. Sequences of the Taqman probes used for SNP genotyping.

SNP Name	Forward Primer(5′–3′)	Reverse Primer (5′–3′)	Probe 1 (VIC)	Probe 2 (FAM)
CAST:c.182A>G (rs109727850)	ACGATGCCCTGGATCAACTTT	TCTCATCTGGATCAGGCTGTCTT	TGCCCGAGAGTGTCAG	TGCCCGAGACTGTCAG
CAST:c.1526T>C (rs109384915)	GCTCCGCCCACAGCA	GAACACTGCTTTCTCAAGACATTTCC	CACTCACCGCTGGAGC	CACTCACCACTGGAGC
CAST:c.1985G>C (rs110914810)	ACGATGCCCTGGATCAACTTT	TCTCATCTGGATCAGGCTGTCTT	TGCCCGAGAGTGTCAG	TGCCCGAGACTGTCAG
CAPN1:c.580A>G (rs17872079)	GCCCAAGGCAACGAGTTCT	GTGGCCTGGAGCTGTCC	TTGGCATAGGCTTTCT	TTGGCATAGGCCTTCT
CAPN1:c.658C>T (rs17872093)	AGCTGCTCCCGCATGTAAG	GGCTGGGCAGGTCAGT	TCCACGCCGTTCCA	CCACGGCGTTCCA
CAPN1:c.948G>C (rs17872000)	CCCCACCCTCTGCAGAGA	GGCAGGGCACGTACCT	CCTGGATCTGGTCATC	CTGGACCTGGTCATC
CAPN1:c.1589G>A (rs17871051)	GCACGTCTGAGGGCTTTGA	TTGCGCAGCTCGTACCA	CACCGGCGGAGTCA	TTCACCGGTGGAGTCA

Supplementary Table S3. Genotype and allele frequencies for 7 selected SNPs in *CAPN1-CAST* Genes.

Marker	Genotype	No. of Animals (n=1000)	Frequency
	GG	342	0.342
CAST:c.182G>A	AG	495	0.495
(rs109727850)	AA	163	0.163
		A	0.411
	AA	349	0.349
CAST:c.1526A>G	AG	477	0.477
(rs109384915)	GG	174	0.174
		G	0.413
	GG	849	0.849
CAST:c.1985G>C	CG	142	0.142
(rs110914810)	CC	9	0.009
		C	0.08
	TT	360	0.36
CAPN1:c.580T>C	CT	473	0.473
(rs17872079)	CC	167	0.167
		C	0.404
	TT	448	0.448
CAPN1:c.658T>C	CC	433	0.433
(rs17872093)	CC	119	0.119
		C	0.336
	GG	407	0.407
CAPN1:c.948G>C	CG	462	0.462
(rs17872000)	CC	131	0.131
		C	0.362
	TT	564	0.564
CAPN1:c.1589T>C	CT	353	0.353
(rs17871051)	CC	83	0.083
		C	0.259
